# Elevated expression levels of androgen receptors and matrix metalloproteinase-2 and -9 in 30 cases of hepatocellular carcinoma compared with adjacent tissues as predictors of cancer invasion and staging

**DOI:** 10.3892/etm.2014.2150

**Published:** 2014-12-19

**Authors:** YAN ZHANG, YUCHENG SHEN, BIN CAO, AITING YAN, HAOMING JI

**Affiliations:** 1 Department of Oncology, Affiliated Hai’an Hospital of Nantong University, Nantong, Jiangsu 226600, P.R. China; 2 Department of Pathology, Affiliated Hai’an Hospital of Nantong University, Nantong, Jiangsu 226600, P.R. China

**Keywords:** hepatocellular carcinoma, androgen receptor, matrix metalloproteinase-2, matrix metalloproteinase-9

## Abstract

The aim of the present study was to investigate the potential roles of the androgen receptor (AR) and matrix metalloproteinase (MMP)-2 and MMP-9 in hepatocellular carcinoma (HCC) tissues and whether their expression could be used as a predictor of the invasion and stage of cancer. The expression levels of AR, MMP-2 and MMP-9 in HCC tissues and tissues adjacent to the tumor were measured by immunohistochemical staining assay. The expression rates of AR, MMP-2 and MMP-9 in the HCC tissue were 76.67, 73.33 and 76.67%, respectively, all of which were significantly higher than those in the tissues adjacent to the tumor. The expression of these proteins represents the local invasion and stage. AR, MMP-2 and MMP-9 expression levels in HCC tissues have the potential to be employed as predictors of the progression of local cancer invasion and the tumor stage.

## Introduction

Hepatocellular carcinoma (HCC) is a common type of malignant tumor in China. The distinguishing characteristics of HCC are its rapid progression and high mortality rate (1). Epidemiological investigation has shown that the morbidity of males with HCC is significantly higher than that of females. According to worldwide statistics, liver cancer in males is the fifth most frequently diagnosed cancer and the second most frequent cause of cancer-related mortality. In females, it is the seventh most commonly diagnosed cancer and the sixth leading cause of cancer mortality (2). The ratio of morbidity in males to that in females is 2–4:1 (3–5). These observations suggest that the occurrence of HCC may be related to the cancer microenvironment, and provide evidence that the androgen receptor (AR) and matrix metalloproteinases (MMPs) may play roles in cancer invasion and potentially lead to the progression of cancer infiltration. The present study was designed to evaluate the possibility that these proteins are significant in cancer invasion and staging by comparing the expression of AR and MMP in HCC tissues with that in tissues adjacent to the HCC.

## Materials and methods

### Origins of specimens

A total of 30 tissue specimens were acquired from the surgical resections of patients with HCC during 2006–2011 in Hai’an Hospital (Nantong, China), which including 26 male cases and 4 female cases. The age range was from 26 to 73 years, with a mean age of 53 years; the median age was 45 years. Of these patients, 22 had undergone palliative resections due to severe hepatic disorders. In 20 cases, the patients had more than one tumor focus, including intrahepatic and distant metastases and/or portal venous tumor emboli (T_3-4_). The remaining 10 cases had only one intrahepatic primary focus (T_1-2_). There were 25 cases (83.33%) that were infected with hepatitis B virus and 17 cases (56.67%) had alcohol-related lesions (Table I). All specimens were confirmed by pathological assay for primary HCC. All cases were selected at the initial diagnosis, and had not been treated with chemotherapy or radiation therapy. All of the patients had never received treatment with sex hormones or steroid hormone drugs. This study was approved by the ethics committee of Hai’an Hospital (Nantong, China). All patients included in the present study provided written informed consent prior to participation.

### Immunohistochemical (IHC) staining

Paraffinized sections (6 μm thick) were prepared from the HCC tissue and peritumoral tissues of every paraffin block specimen. All paraffin sections were analyzed by immunohistochemical methods using AR, MMP-2 and MMP-9 assay kits (Boster Biological Engineering Co., Ltd., Wuhan, China). A prostate tissue section was used as the positive control for AR expression, while phosphate-buffered saline (PBS) was instead of the primary antibody to treat the section used as an internal negative control.

### Judgment standards

Every immunostained section was analyzed by two independent pathologists. Cytoplasmic and/or or nuclear staining (brown reaction products) was regarded as a positive result. The sections were viewed under a microscope and 10 high power field were randomly selected. ARs were located in the nuclei of HCC cells and the tissue adjacent to the carcinoma. Points were awarded according to the percentage of stained cells in the tumor and peritumoral tissue, and were divided into following categories: 0%, 0 points; 1–9%, 1 point and >10%, 2 points. Points were also awarded for staining intensity, and were divided into the following categories: No staining, 0 points; weak staining (buff in color), 1 point; medium staining (brown-yellow), 2 points; and strong staining (brown), 3 points. The two types of points were added together, and a score >2 was recorded as AR expression positive; otherwise, AR expression was regarded as negative. MMP-2 and MMP-9 were located in the cytoplasm of HCC cells and the peritumoral cells. Points were awarded according to the percentage of stained cells in the tumor and peritumoral tissue as follows: 0%, 0 points; <25%, 1 point; 25–49%, 2 points; and >50%, 3 points. Points were also awarded for staining intensity as follows: No staining, 0 points; weak staining (buff in color), 1 point; medium staining (brown-yellow), 2 points; and strong staining (brown), 3 points. The two types of points were added together, and a score >3 was recorded as MMP-2 or MMP-9 expression positive; otherwise, the expression was regarded as negative.

### Statistical analysis

Data analysis was performed using the χ^2^ test with SPSS software, version 13.0 (SPSS Inc, Chicago, IL, USA) for Windows. P<0.05 was considered to indicate a statistically significant result.

## Results

### AR, MMP-2 and MMP-9 expression

The expression levels of AR, MMP-2 and MMP-9 in the HCC tissues and adjacent tissues are presented in Table II. The positive expression rates of AR, MMP-2 and MMP-9 in the HCC tissues were higher than those in the tissues adjacent to the HCC (P<0.05). The expression levels of AR, MMP-2 and MMP-9 were significantly different between the T_3_/T_4_ and T_1_/T_2_ stages of HCC (P<0.05, Table III).

**Figure 1:**
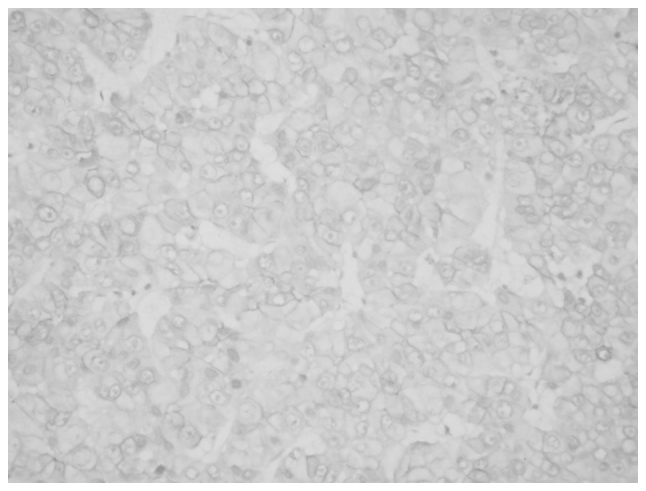
Immunostained section of peritumoral tissue. Magnification, ×40.

**Figure 2:**
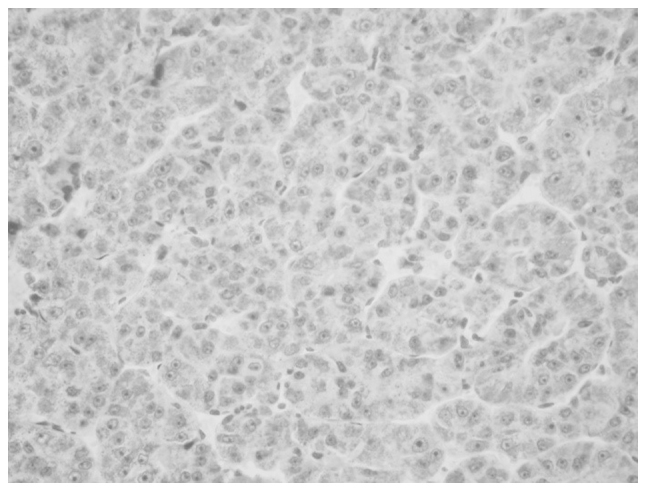
Immunostained section of hepatocellular carcinoma tissue. Magnification, ×40.

*Immunostained sections:* Figs. 1 shows the representative immunostained sections of peritumoral tissue and Fig. 2 shows the AR(+) HCC tissue from the specimens analyzed in the present study. The MMP-2 or MMP-9 expression in the immunohistochemical staining sections was similar to the AR expression results.

## Discussion

HCC is a kind of highly invasive malignant tumor, which is always diagnosed according to clinic symptoms and signs, imaging data and specific markers such as α-fetoprotein (6). Once it occurs, HCC always progress rapidly and only a few cases can be diagnosed at an early stage. Factors associated with the prognosis index of HCC include diagnosis stage, tumor size, the completeness of the tumor capsule, vascular invasion, pathological type and cell proliferation (7,8). The majority of studies have shown AR expression in HCC tissues (9,10,11). However, due to the specimens being from different sources, such as fresh specimens and older paraffin-embedded specimens, differences in the detection methods, variations in the sample sizes and racial differences in the patients, the positive rate of the AR in different reports is variable. The present study found that the positive rate of AR was 76.67%, which was similar to that in the majority of the reports. The study also confirmed that the positive rate of AR expression in primary liver cancer cells is significantly higher than that in adjacent cells (P<0.05). Subgroup analysis according to stage also indicated that the positive rate of AR expression in HCC patients with intrahepatic metastasis, distant metastasis or portal vein invasion was higher than that in patients without any metastasis, and the difference was significant (P<0.05). This finding indicated that the positive expression of AR in HCC is associated with vascular invasion of the liver and metastasis. Males under 50 years of age comprised 76.7% of the study group (n=30); the data shows that young male patients were in the majority in this group of HCC patients at the first diagnosis, and that their tumors invade and transfer earlier. The present study demonstrated that androgens and the androgen-AR signaling pathway in patients may play a very important role in the biological behavior of HCC, such as in its occurrence, development, invasiveness and metastasis. A previous study found that ARs are able to adjust hepatitis B virus transcription and promote the formation of HCC associated with hepatitis B (9); however, the mechanism by which androgens and the androgen-AR signaling pathway regulate and control hepatocarcinogenesis and its progress are unclear. The majority of primary liver cancer patients have hepatitis B virus infection or lesions due to chronic alcohol use. The metabolism of androgens has been observed to be decreased or disordered in patients when chronic hepatic lesions are present (12). It is unclear whether hepatocarcinogenesis and its progress are associated with long-term and continued high levels of androgens. Further studies concerning the association between the androgen-AR signaling pathway and HCC are required.

Invasion and metastasis of a tumor are multiple and complicated processes. Cancer cells break away from the primary lesion and initially break through the extracellular matrix and basement membrane. Degradation of the extracellular matrix and the basement membrane is one pivotal step. Extracellular matrix degradation occurs due to the actions of matrix metalloproteinases, hyaluronidase and a large number of phagocytic cells in liver, and MMP-2 and MMP-9 enzymes play important roles in the degradation of the extracellular matrix and basement membrane. MMP-2 and MMP-9 are not only involved in the enzymolysis of intercellular matrix components, but also are the key enzymes in the degradation of extracellular matrix collagen IV (13). MMP-2 and MMP-9 exist extensively in HCC tissues. The results of the present study indicate that MMP-2 and MMP-9 are expressed at higher levels in HCC tissue than in peritumoral tissues. Subgroup analysis according to stage suggests that the rate of distant metastases or portal vein invasion in patients that are MMP-2 or/and MMP-9 positive is significantly higher than that of patients who are negative for these enzymes (P<0.05), which is consistent with previous studies (14,15). The results of the present study confirmed that the overexpression of MMP-2 and MMP-9 is likely to increase tumor invasion and metastasis.

In summary, ARs are associated with local cancer cell infiltration, in which MMP-2 and MMP-9 may play important roles in the HCC microenvironment to increase invasion and metastasis (16). AR, MMP-2 and MMP-9 may be predictive markers for HCC and could be regarded as candidates for indicating the cancer stage.

## Figures and Tables

**Table I:** Clinical characteristics of 30 patients with HCC.

Clinical feature	No. of cases
Gender	
Male	26
Female	4
Age (years)	
<50	23
≥50	7
Tumor number	
One	10
Multiple	20
Size of tumor	
<5 cm	10
≥5 cm	20
Hepatitis B virus	
Yes	25
No	5
Alcohol drinker	
Yes	17
No	13

HCC, hepatocellular carcinoma.

**Table II:** Expression of AR, MMP-2 and MMP-9 in HCC tissues and tissues adjacent to HCC.

Tissue	n	AR (+)	MMP-2 (+)	MMP-9 (+)
HCC	30	23	22	23
Adjacent to carcinoma	30	8	13	15
χ^2^		15.017	5.554	4.593
P-value		0.000	0.018	0.032

AR, androgen receptor; MMP, matrix metalloproteinase; HCC, hepatocellular carcinoma.

**Table III:** Expression of AR, MMP-2 and MMP-9 in different stages of HCC.

Stage	n	AR (+)	MMP-2 (+)	MMP-9 (+)
T_3_/T_4_	20	18	17	19
T_1_/T_2_	10	5	5	4
χ^2^		5.963	4.176	11.273
P-value		0.015	0.041	0.001

AR, androgen receptor; MMP, matrix metalloproteinase; HCC, hepatocellular carcinoma.
